# Spontaneous spinal epidural hematoma in a patient on acenocoumarol for valvular atrial fibrillation: A rare case report

**DOI:** 10.1016/j.amsu.2021.103076

**Published:** 2021-11-17

**Authors:** Mohammed El-azrak, Mohammed Noumairi, Mohammed Amine Oulalite, Siham El Mir, Safaa Kachmar, Houssam Bkiyar, Noha El Ouafi, Ahmed Amine El Oumri, Zakaria Bazid, Brahim Housni

**Affiliations:** a Department of Cardiology, Mohammed VI University Hospital, Faculty of Medicine and Pharmacy of Oujda, Mohammed First University, Oujda, Morocco; b Department of Physical Medicine and Rehabilitation, Mohammed VI University Hospital, Faculty of Medicine and Pharmacy of Oujda, Mohammed First University, Oujda, Morocco; c Department of Anesthesiology and Reanimation, Mohammed VI University Hospital, Faculty of Medicine and Pharmacy of Oujda, Mohammed First University, Oujda, Morocco; d Epidemiological Laboratory of Clinical Research and Public Health, Faculty of Medicine and Pharmacy of Oujda, Mohammed First University, Oujda, Morocco

**Keywords:** Spontaneous spinal epidural hematoma, Vitamin K antagonists, Atrial fibrillation, Paraplegia, Sphincter disturbance, Rehabilitation

## Abstract

**Introduction:**

Spontaneous spinal epidural hematoma (SSEH) is a rare finding, but one with serious clinical implications. Oral anticoagulant drugs are known to be associated with the SSEH onset, particularly when combined with drugs increasing the bleeding risk.

**Case presentation:**

We present the case of a 62-year-old female on acencoumarol for her atrial fibrillation complicating severe mitral stenosis with a history of Ketoprofen use for the onset of her first symptoms. She presented to our emergency room with paraplegia and sphincter disturbance. Spinal magnetic resonance imaging (MRI) revealed a posterior SSEH extended from T10 to T12 requiring an urgent decompression of the spinal cord by laminectomy performed within 48 hours from the symptom's onset. After 3 months of rehabilitation, the patient improves partially her muscular strength with mostly unchanged sensitive and sphincteric levels.

**Clinical discussion:**

Vitamin K antagonists (VKA) use appears to be a high suspicion index for SSEH diagnosis resulting in earlier surgery and improving neurological outcome. Also, it is important to pay attention to the concomitant use of VKA and non-steroidal anti-inflammatory drugs which increase the risk of bleeding and may worsen the neurological outcome.

**Conclusion:**

SSEH is a rare and serious finding which should be especially searched when a history of oral anticoagulation is reported in presence of neurological symptoms. A prompt and suitable management may improve the patient outcomes.

## Highlights

• Spontaneous spinal epidural hematoma (SSEH) is a rare neurological emergency with serious clinical implications.
• Oral anticoagulants may be associated with SSEH, particularly when combined with drugs increasing bleeding risk.
• MRI is the standard of choice for diagnosing SSEH and early surgical laminectomy with hematoma evacuation is recommended.

## 1 Introduction

Spontaneous spinal epidural hematoma (SSEH) is a non-traumatic and non-iatrogenic neurological emergency due to an accumulation of blood in the epidural space [1,2]. Its incidence is of 1 per 1000000 persons (1). It occurs mainly in the elderly with a male/female ratio of 1.4/1 [3]. Some studies reported that upwards 17–30% of SSEH cases are found to be associated with a history of oral anticoagulants use [4,5] and the combined use of non-steroidal anti-inflammatory drugs (NSAID), even transient or prolonged predispose to a real risk of bleeding [6].

Symptoms can range from a pain in the neck or the back, to complete neurological deficits, according to the site and the pattern of spinal compression [7]. The diagnosis is often delayed, even if improved by the generalization of magnetic resonance imaging [8].

Most patients diagnosed with SSEH undergo an early surgical spinal decompression consisted of a laminectomy with hematoma evacuation. In selective patients, some scholars preconise a conservative treatment [4,9].

In this article, we report a case of a SSEH in a patient on vitamin K antagonists (VKA) for her valvular atrial fibrillation using transiently a NSAID resulting in a complete spinal injury. We hope this will help to raise awareness about the particular precaution must be attributed to VKA drug interaction which can lead dramatically to severe neurological deficits.

This case report has been reported in line with the SCARE 2020 criteria [10].

## 2 Case presentation

A 62-year-old female presented to our emergency department with paraplegia of the lower limbs and sphincter disturbance. Her past medical history included arterial hypertension, atrial fibrillation (AF) complicating a severe mitral valve stenosis for which she is on 2mg per day of acenocoumarol for 10 years with no drug history, family history, or psychosocial history. Her other daily medications were: Aldactone 50mg/day, Carvedilol 6.25mg/day and Furosemide 40mg/day. The patient experienced one week before her presentation a non-traumatic low back pain for which she auto medicates herself with Ketoprofen 100mg b.i.d for two consecutive days. She next developed neurological signs consisting of numbness making it difficult to walk and tingling sensation of both lower limbs. In two days, she was uncapable of walking, could not feel her limbs and complained about constipation and acute urinary retention, which brought her to our emergency room.

On presentation the patient's vital signs were within normal limits, with an arterial pression of 150 mmHg/80 mmHg, a heart pulse of 95 beats per minute, an oxygen saturation of 100% on room air. On clinical examination, the patient was alert and oriented to time and space, with irregular heartbeats and a diastolic murmur of mitral stenosis on auscultation. The neurological exam has shown paraplegia with muscular strength of the right and left lower limbs to be 1/5 which are hypotonic, anesthesia more accentuated on the left side, abolished deep tendon reflexes with a T10 clinical lesion level classified A on American spinal cord injury (ASIA) impairment scale. Blood examinations did not reveal any particular findings and INR was under the therapeutic range for her AF. Significant results are shown on Table 1.

**Table 1:** Significant laboratory findings.

Test	Results	Normal range
Albumin	42.00	38–50g/L
C -Reactive Protein	8.96	<12mg/L
Urea	0.54	0,1–0,5 g/L
Prothrombin	65	(70–100%)
INR	1,6	**2–3**
WBC	10,7	**4.0**–**10.5 x 103/uL**
Hemoglobin	14.5	**12.1**–**15.8 g/dL**
Platelets	199	(150 - 400 **x 103/μL**)

WBC, white blood cell; INR, international normalized ratio.

**Fig. 1:**
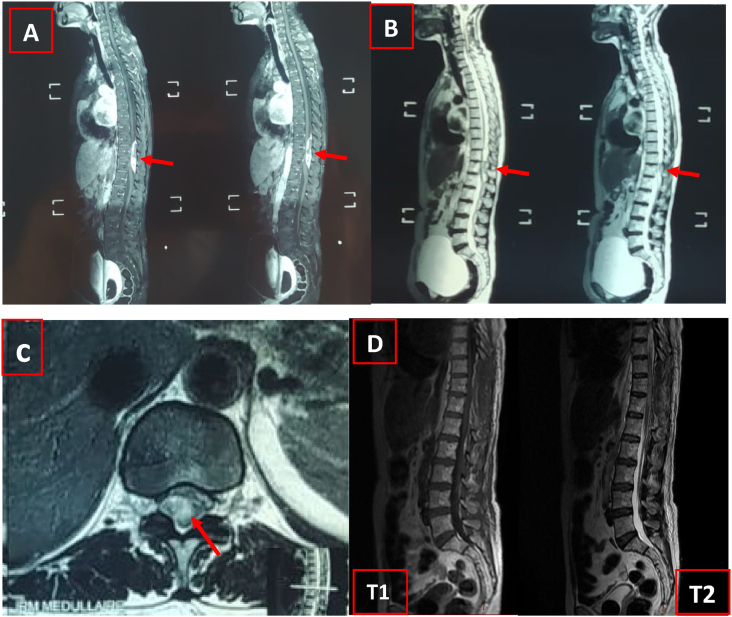
**(A)** T1 weighted Magnetic resonance imaging in sagittal view demonstrating spontaneous spinal epidural hematoma (SSEH) from T10 to T12 indicated by the arrow. **(B)** T2 weighted MRI images in sagittal view indicating SSEH (Arrows) **(C)** T2 weighted MRI images in transversal view showing the SSEH (Arrow) **(D)** T1 et T2 weighted MRI images performed after surgery showing the complete evacuation of hematoma.

An urgent cervical and thoracolumbar spinal MRI was performed revealing a spontaneous posterior epidural hematoma extended from T10 to T12 with an important spinal compression at this level. T2-weighted images didn't show spinal suffering signs (Fig. 1: A-C). As the patient was on acenoucomarol, a probabilistic single dose of 25 IU/kg of Prothrombin complex concentrates and 10 mg of vitamin K were given as soon as the diagnosis was made. The patient was then assessed by a neurosurgeon and brought timely to the operating room where she underwent a decompressive laminectomy of T10-T12 made by a professor of neurosurgery with the aid of a junior trainee with 4 years of surgical specialty training. No neurological improvement was noticed after surgery and MRI repeated 2 days after has shown a complete resolution of the SSEH and no spinal cord abnormalities (Fig. 1: D).

The patient was discharged a week later and addressed to the physical medicine and rehabilitation department for an adapted rehabilitation program with a urodynamic assessment. After 3 months of rehabilitation, we found an improvement in motor strength becoming 3/5 in both lower limbs with mostly unchanged sensitive and vesico-sphincter levels; ASIA impairment scale C. The patient still benefiting from a home rehabilitation program.

## 3 Discussion

SSEH is a rare neurological affection described for the first time by Jackson in1869. The etiology is still uncertain. Coagulopathies, anti-coagulant therapy, malignancy and pregnancy are often reported in the presence of SSEH [11,12]. Some researchers suggested the degenerative origin of vessels to be the etiology [12]. Clearly, Spinal epidural arteriovenous fistula leads to congestion of the spinal epidural venous plexus which degenerate progressively leading finally to a weak venous plexus. An increasing intra thoracic or intra-abdominal pressure may then cause the rupture of the vessel's walls and the formation of a spinal epidural hematoma [13]. Furthermore, Miyagi et al. thought of an arterial source because the lower pressure in the internal vertebral venous plexus compared to that of epidural space [14].

Oral anticoagulants use is associated with 17–30% of SSEHs [4,5]. It is a serious hemorrhagic complication that involves the vital prognosis. To be avoided, the anticoagulant intensity of VKA should be regularly assessed by International Normalized Ratio (INR), which depends on patient observance, acute medical conditions, comorbidities and medical drug interactions [15]. Nevertheless, no absolute ‘safe’ INR is to be considered because many published cases of SSEH were in the therapeutic range [16]. In our case, the patient used acenocoumarol; widely prescribed in our country, for the prevention of thromboembolic risk associated with AF complicating her severe mitral stenosis with an admission INR of 1.6. She potentialized the bleeding risk and worsened the neurological symptoms by taking Ketoprofen for the onset of her first symptoms. The mechanism of NSAIDs-induced bleeding is probably the reduced production of thromboxane A2 by inhibiting cyclooxygenase-1 leading to platelet aggregation inhibition [17].

Symptoms of SSEH are typically initiated by neck or back pain occurring occasionally or permanently, preceding neurological deficits onset of variable severity degree including bladder and bowel disorders. Complete spinal injury can take few hours to many days to appear, which is the case in patients with lumbar SSEH [18].

MRI is the diagnostic tool of choice for SSEH. The sagittal coupe shows typically a spindle shape image with a central maximal compression commonly located at the C6 and/or Th12 levels. Hematoma within the first 24 hours of symptom onset is isointense on T1-weighted images and can sometimes last many days after. Hematoma acquires a hyperintense signal on T1 and T2-weighted images after 36h [19].

Once the diagnosis made, early surgical laminectomy with hematoma evacuation should be performed with a tailored dural opening if MRI is uncapable to precise the exact bleeding site. Surgery is more effective when done within 12–36 hours from the symptoms onset and recovery is faster [3,20]. As reported in many studies, in patients with mild symptoms and rapid clinical improvement, conservative treatment can be adopted with good outcomes [9,21]. In these studies, 30% of patients had residual neurological deficits. Moreover, it was reported that the hematoma can be evacuated efficiently by full-endoscopic transforaminal approach with minimal invasiveness [22].

Determining factors of prognosis include interval of symptoms onset, operative interval, pre therapeutic ASIA score reflecting the severity of neurological deficit, hematoma size and location with worse outcome in thoracic site and the number of concerned segments [3,13]. In addition, spinal cord edema visualized on MRI was reported to be correlated with poor prognosis [23].

Our patient exhibited bilateral paraplegia, complete sensitive extinction, bladder and bowel dysfunction with ASIA impairment scale (A); hence, the prognosis was poor. She was discharged with a rehabilitation program started a week later with a satisfying motor improvement and no sensitive improvement 3 months later. ASIA impairment scale was C at the end of the program.

## 4 Conclusion

Spontaneous spinal epidural hematoma is a rare and serious neurological emergency of the spinal cord which should be suspected in the presence of a history of oral anticoagulation, especially when combined with another interacting drug in a patient with neurological deficits. An urgent MRI imaging must be performed and once the diagnosis made, surgery within the 48 hours after symptoms onset must be undergone after a detailed neurological assessment.

## Ethical approval

The ethical committee approval was not required give the article type (case report).

## Sources of funding

This research did not receive any specific grant from funding agencies in the public, commercial, or not-for-profit sectors.

## Author contribution

Mohammed El-azrak: Study concept, data analysis, writing the paper; Mohammed Noumairi and Safaa Kachmar: data analysis; Mohammed Amine Oulalite and Siham El Mir; Houssam Bkiyar, Ahmed Amine El Oumri, Noha El Ouafi, Zakaria Bazid and Brahim Housni: Conception, methodology, supervision.

## Consent

Written informed consent was obtained from the patient for publication of this case report and accompanying images. A copy of the written consent is available for review by the Editor-in-Chief of this journal on request.

## Registration of Research Studies

Name of the registry:

Unique Identifying number or registration ID:

Hyperlink to your specific registration (must be publicly accessible and will be checked):

## Guarantor

MOHAMMED EL-AZRAK.

## Provenance and peer review

Not commissioned, externally peer-reviewed.

## Declaration of competing interest

No conflicts are declared.
